# Inter-patient variations in relative biological effectiveness for cranio-spinal irradiation with protons

**DOI:** 10.1038/s41598-020-63164-8

**Published:** 2020-04-10

**Authors:** Kristian S. Ytre-Hauge, Lars Fredrik Fjæra, Eivind Rørvik, Tordis J. Dahle, Jon Espen Dale, Sara Pilskog, Camilla H. Stokkevåg

**Affiliations:** 1 Department of Physics and Technology, University of Bergen, Bergen, Norway; 2 Department of Medical Physics, Oslo University Hospital, The Radium Hospital, Oslo, Norway; 3 Department of Oncology and Medical Physics, Haukeland University Hospital, Bergen, Norway

**Keywords:** Cancer therapy, CNS cancer, Paediatric cancer, Physics

## Abstract

Cranio-spinal irradiation (CSI) using protons has dosimetric advantages compared to photons and is expected to reduce risk of adverse effects. The proton relative biological effectiveness (RBE) varies with linear energy transfer (LET), tissue type and dose, but a variable RBE has not replaced the constant RBE of 1.1 in clinical treatment planning. We examined inter-patient variations in RBE for ten proton CSI patients. Variable RBE models were used to obtain RBE and RBE-weighted doses. RBE was quantified in terms of dose weighted organ-mean RBE (${\overline{{\rm{RBE}}}}_{{\rm{d}}}$ = mean RBE-weighted dose/mean physical dose) and effective RBE of the near maximum dose (D_2%_), i.e. RBE_D2%_ = ${D}_{2 \% ,RBE}/{D}_{2 \% ,phys}$, where subscripts *RBE* and *phys* indicate that the D_2%_ is calculated based on an RBE model and the physical dose, respectively. Compared to the median ${\overline{{\rm{RBE}}}}_{{\rm{d}}}$ of the patient population, differences up to 15% were observed for the individual ${\overline{{\rm{RBE}}}}_{{\rm{d}}}$ values found for the thyroid, while more modest variations were seen for the heart (6%), lungs (2%) and brainstem (<1%). Large inter-patient variation in RBE could be correlated to large spread in LET and dose for these organs at risk (OARs). For OARs with small inter-patient variations, the results show that applying a population based RBE in treatment planning may be a step forward compared to using RBE of 1.1. OARs with large inter-patient RBE variations should ideally be selected for patient-specific biological or RBE robustness analysis if the physical doses are close to known dose thresholds.

## Introduction

Proton therapy has been established as an important radiation treatment modality for cancer, offering improved sparing of normal tissue compared to conventional radiation therapy with photons^1^. The dosimetric advantages of protons have already been shown for medulloblastoma patients receiving cranio-spinal irradiation (CSI)^2,3^, and early clinical outcomes suggest that similar disease control can be achieved with reduced toxicity compared to photon therapy^4–6^. Longer follow-up and more clinical data are awaited to draw firm conclusions on the clinical benefits of proton CSI^7^.

Dosimetric benefits of proton CSI include a significant dose reduction to organs anterior to the spinal column, including the cochlea, transverse colon, stomach, kidney, heart, and lungs^8^. Besides the different spatial patterns in physical dose deposition, protons also have an increased biological effect compared to photons, i.e. protons produce more damage than photons from the same physical dose. This difference is currently accounted for by a dose scaling factor; the relative biological effectiveness (RBE). Although contemporary clinical proton treatment planning is based on a constant RBE of 1.1 (RBE_1.1_), the proton RBE is known to vary with linear energy transfer (LET), tissue type and physical dose^9–11^. In particular, the RBE increases due to the increase in LET towards the end of the proton beam range. The need to investigate the RBE further and to and to provide clinical solutions accounting for variations in the RBE has therefore recently been emphasized by several research groups^10,12,13^. To quantify RBE variations in proton therapy, several so-called variable RBE models have been developed. These models are commonly based on analysis of empirical data from *in vitro* proton and photon irradiation of multiple cell lines and use the linear-quadratic model with cell inactivation as a biological endpoint to estimate the RBE^14^. Due to the spatial variations in RBE within the patient, the biologically effective dose distribution received during proton CSI may differ significantly from dose estimates based on the constant RBE of 1.1, especially for organs at risk (OARs) located distal to the target volume, where the LET, and therefore RBE, typically is high^12^. Although the RBE is elevated, the strong decrease in physical dose beyond the proton Bragg peak leads to a significant potential for sparing OARs compared to photon-based CSI.

For passively scattered (PS) proton CSI treatments, Giantsoudi *et al*.^15^ studied the LET and RBE with emphasis on RBE-weighted doses (RBE × dose) to the brainstem using two different variable RBE models, and reported mean RBE estimates consistently above 1.1 in the brainstem. Further, the RBE-weighted doses to OARs in two CSI patients were investigated in a study of vertebral column sparing techniques by applying five different treatment planning strategies^16^. This study showed elevated RBE for OARs distal to the spine, including the thyroid, lungs, esophagus and heart, and that the RBE values and doses were strongly dependent on the target volume delineation. The proton RBE for the target volumes of medulloblastoma patients was also assessed by Jones *et al*.^17^ who suggested a risk of tumor under-dosage due to indications of low RBE in medulloblastoma tumor cells compared to surrounding tissues. The same study also emphasizes the risks from elevated RBE in brain tissues.

While previous studies have investigated inter-patient variations in RBE for several other indications^18–21^, the RBE variations in standardized CSI proton treatments have so far received little attention. For CSI using intensity modulated proton therapy (IMPT), the variations in RBE in OARs across different patients and RBE models remains unexplored, and may differ from those found for PS protons^22^. The objective of our study was therefore to investigate inter-patient variations in RBE for patients receiving CSI with IMPT, assessing also the impact of uncertainties in tissue parameters and differences between RBE models. Two variable RBE models, based on a broad range of *in vitro* experiments, were applied to calculate the RBE and RBE-weighted dose for ten CSI patients. The (α/β)_x_ (fractionation sensitivity of photon-based radiotherapy), LET and (physical) dose were used as model input parameters. The LET and dose were obtained through Monte Carlo simulations on a voxel-by-voxel basis for each patient and treatment plan.

## Results

**Figure 1:**
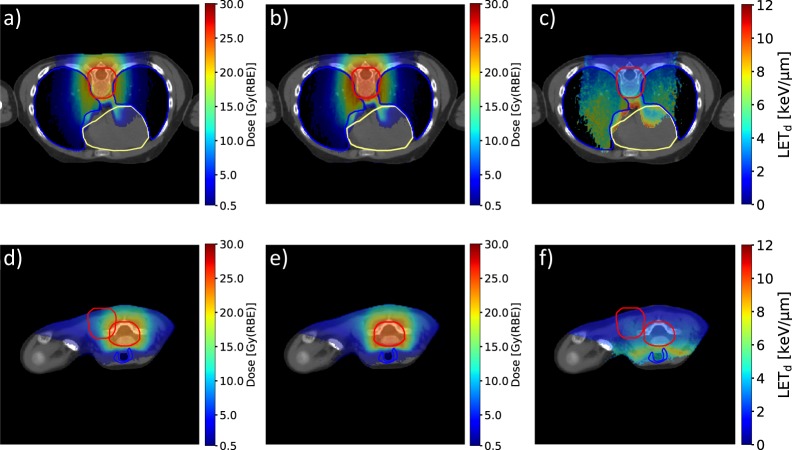
Dose distributions obtained with RBE_1.1_ (**a**,**d**) and the variable RBE model ROR ((α/β)_x_ of 3.0 Gy) (**b**,**e**), and the corresponding LET_d_ distribution (**c**,**f**). Dose and LET_d_ values in voxels receiving less than 0.5 Gy(RBE) are set transparent. The spinal planning target volume, lungs, heart and thyroid are outlined in red, blue, yellow and blue, respectively.

Dose and dose-averaged LET (LET_d_) distributions for one patient are shown in Fig. 1. The lungs, heart and thyroid are partially exposed to moderate doses and relatively high LET_d_ values. Compared to the RBE_1.1_ doses (Fig. 1a,c) the RBE-weighted doses from the variable RBE model by Rørvik *et al*. (ROR)^9^ (Fig. 1b,e) are slightly elevated, indicating RBE above 1.1. A clear increase in LET_d_ can be seen along the beam direction (Fig. 1c,f). Further comparison to a photon therapy treatment plan for this patient can be found in Fig. 1S and 2S in the Supplementary Materials.

**Figure 2:**
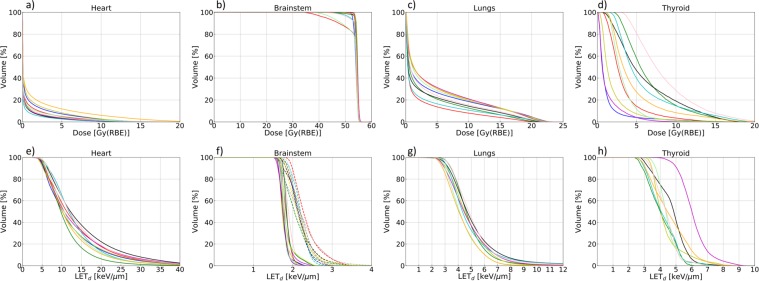
The upper panels show DVHs for the heart (**a**), brainstem (**b**), lungs (**c**) and thyroid (**d**) for all ten patients calculated with RBE_1.1_. The lower panels show LVHs for the heart (**e**), brainstem (**f**), lungs (**g**) and thyroid (**h**). For the brainstem, LET_d_ for the CSI and boost plans are plotted separately with solid and dashed lines, respectively.

Dose volume histograms (DVHs) using RBE_1.1_ and LET volume histograms (LVHs) for the OARs are shown in Fig. 2, illustrating the degree of inter-patient variations for different OARs in physical dose and LET_d_ which both affects the RBE. Dose, LET_d_, and RBE metrics are reported in Table 1 for all OARs and RBE models. The greatest variation in dose between patients was seen for the thyroid, with mean doses in the range 1.2–8.4 Gy(RBE) for RBE_1.1_ which increased to 1.7–10.5 Gy(RBE) using the variable RBE model by McNamara *et al*.^23^ (MCN). Variations in LET_d_ across the patients were greatest for the thyroid and heart. The inter-patient variations in near maximum dose (D_2%_) were also largest for the thyroid and heart, and again, the variable RBE models gave higher doses compared to RBE_1.1_.

**Table 1:** Dose, RBE and LET_d_ metrics reported as median and range over the patient group for the different RBE models and OARs.

Structure	${\overline{{\rm{LET}}}}_{{\rm{d}}}$ median (Range) [keV/µm]	RBE model	D_50_ median (Range) [Gy(RBE)]	D_2%_ median (Range) [Gy(RBE)]	${\overline{{\rm{RBE}}}}_{{\rm{d}}}$ median (Range)	RBE_2%_ median (Range)
		***RBE***_***1.1***_	0.7 (0.4–1.7)	8.5 (6.0–16.0)	—	—
**Heart**	13.0 (9.6–15.0)	***MCN***_***3.0Gy***_	0.9 (0.6–2.4)	11.6 (8.5–20.3)	1.62 (1.53–1.65)	1.51 (1.40–1.56)
		***ROR***_***3.0Gy***_	0.9 (0.6–2.3)	11.2 (8.2–19.6)	1.56 (1.47–1.59)	1.46 (1.35–1.51)
		***RBE***_***1.1***_	54.3 (52.8–54.6)	55.4 (55.1–55.6)	—	—
**Brainstem**	1.8 (1.8–1.9) (CSI)	***MCN***_***2.1Gy***_	58.1 (56.8–58.5)	59.8 (59.3–60.4)	1.18 (1.17–1.18)	1.18 (1.18–1.20)
	2.2 (2.1–2.3) (boost)	***ROR***_***2.1Gy***_	56.5 (55.2–56.8)	57.9 (57.5–58.3)	1.14 (1.14–1.15)	1.15 (1.14–1.16)
		***RBE***_***1.1***_	4.0 (2.2–4.8)	20.4 (17.6–21.4)	—	—
**Lungs**	4.8 (3.9–5.2)	***MCN***_***4.0Gy***_	4.7 (2.5–5.6)	22.6 (19.6–23.6)	1.27 (1.24–1.29)	1.22 (1.20–1.23)
		***ROR***_***4.0Gy***_	4.6 (2.4–5.5)	22.1 (19.2–23.1)	1.25 (1.23–1.27)	1.20 (1.18–1.20)
		***RBE***_***1.1***_	3.9 (1.2–8.4)	14.0 (6.2–18.7)	—	—
**Thyroid**	4.5 (4.1–6.1)	***MCN***_***3.0Gy***_	5.2 (1.7–10.5)	17.6 (9.4–22.2)	1.44 (1.36–1.67)	1.40 (1.31–1.66)
		***ROR***_***3.0Gy***_	5.1 (1.7–10.3)	17.1 (9.0–21.6)	1.42 (1.34–1.61)	1.36 (1.27–1.58)

Results for the variable RBE models are reported for the nominal (α/β)_x_ values, indicated by subscripts in the table.

**Figure 3:**
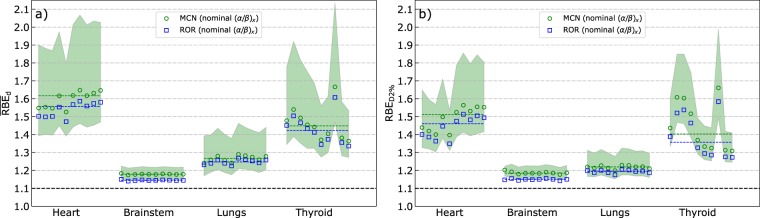
Mean dose-weighted RBE (**a**) and effective near maximum dose RBE (**b**) for the heart, brainstem, lungs and thyroid in the ten patients. The markers show the RBE values for individual patients, while the dashed lines represent the median over the patient group. The shaded region represents the possible RBE values for the MCN model within the (α/β)_x_ confidence intervals for the different OARs.

Figure 3 shows the calculated dose-weighted organ-mean RBE values (${\overline{{\rm{RBE}}}}_{{\rm{d}}}$) and effective near maximum dose RBE (RBE_D2%_) for all patients and OARs, as well as the median ${\overline{{\rm{RBE}}}}_{{\rm{d}}}$ and median RBE_D2%_ across the patient population for each RBE model, calculated from the ten individual ${\overline{{\rm{RBE}}}}_{{\rm{d}}}$ and RBE_D2%_ values, respectively. The inter-patient variations in ${\overline{{\rm{RBE}}}}_{{\rm{d}}}$ and RBE_D2%_, follow the same trends: Large inter-patient RBE variations were observed for the thyroid, followed by moderate variations for the heart and lungs, whereas only minor variations were seen for the brainstem, with maximum deviation from the median ${\overline{{\rm{RBE}}}}_{{\rm{d}}}$ and RBE_D2%_ of 0.5% and 1.5%, respectively. In comparison, the difference in brainstem ${\overline{{\rm{RBE}}}}_{{\rm{d}}}$ between the ROR and MCN models was between 2.8% and 3.0% for all patients, with the MCN model resulting in the highest values. In general, the ROR and MCN models predicted similar RBE values across the patient population, but with slightly higher values for the MCN model. The maximum absolute difference in ${\overline{{\rm{RBE}}}}_{{\rm{d}}}$ and RBE_D2%_ between the models was 0.07 and 0.09, respectively. For the heart and lungs, slightly lower RBE_D2%_ values were observed compared to the ${\overline{{\rm{RBE}}}}_{{\rm{d}}}$, while no consistent differences between RBE_D2%_ and ${\overline{{\rm{RBE}}}}_{{\rm{d}}}$ were observed for the thyroid and brainstem.

The RBE variations were largest in the organs with inhomogeneous dose distributions, and the inter-patient variations in ${\overline{{\rm{RBE}}}}_{{\rm{d}}}$ for the thyroid (up to 15%) may be attributed to the wide range in dose and in LET between patients (Fig. 2d,h). The variations in RBE within the (α/β)_x_ confidence intervals (CI) were larger than both inter-patient variations and model differences, with the lowest (α/β)_x_ values resulting in the highest RBE, as expected.

**Figure 4:**
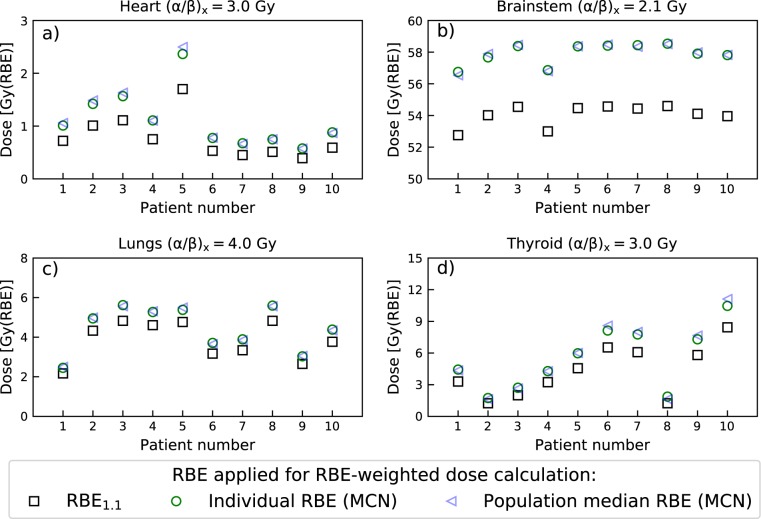
Comparison of RBE-weighted mean doses for the heart (**a**), brainstem (**b**), lungs (**c**) and thyroid (**d**) calculated from the population median RBE (blue), the patient specific (individual) RBE (green) and RBE_1.1_ (black). RBE estimates are based on the MCN model and nominal (α/β)_x_ values.

The population median ${\overline{{\rm{RBE}}}}_{{\rm{d}}}$ values for the MCN model were 1.62 (heart), 1.18 (brainstem), 1.27 (lungs) and 1.44 (thyroid). These values differ significantly from the clinically applied RBE of 1.1, but are only slightly different from most individual ${\overline{{\rm{RBE}}}}_{{\rm{d}}}$ values across the ten patients, as can be seen by comparing the data points and dashed lines in Fig. 3a. The clinical impact of the RBE variations are, however, dependent on the physical dose levels which varies strongly between the OARs. The resulting inter-patient variations in RBE-weighted doses and how these differ from doses based on RBE_1.1_ are illustrated in Fig. 4. The figure shows RBE-weighted doses calculated for each of the ten patients using both their individual RBE (data points in Fig. 3a) and the population median ${\overline{{\rm{RBE}}}}_{{\rm{d}}}$ (dashed lines from Fig. 3a), together with RBE-weighted doses calculated with RBE_1.1_. The variable RBE doses are calculated using the MCN model with nominal (α/β)_x_ values, and the largest inter patient variation in RBE-weighted dose are again seen for the thyroid. However, we can also see that the largest deviations in RBE-weighted dose compared to RBE of 1.1 are present for the brainstem, mainly due to the high physical dose here. Overall, it is clear that the population based dose estimates are much closer to the individually calculated RBE-weighted doses as compared to the RBE_1.1_ doses. This is particularly evident for the brainstem. The largest deviation from the population median ${\overline{{\rm{RBE}}}}_{{\rm{d}}}$ was 0.22 (thyroid of patient number 8). The mean dose to the thyroid for this patient (calculated with RBE_1.1_) was, however, only 1.24 Gy(RBE). The difference in RBE-weighted dose calculated from the population median RBE and the patient specific RBE was therefore only 0.25 Gy(RBE). However, for the RBE_D2%_, the corresponding difference was 1.46 Gy(RBE). For most cases, the difference in RBE-weighted dose calculated with the individual and median RBE was negligible, with maximum differences (in Gy(RBE)) of 0.14 (heart), 0.22 (brainstem), 0.11 (lungs), 0.65 (thyroid). Similar results were also seen for the ROR model.

## Discussion and conclusion

Overall, the largest inter-patient variations in organ specific RBE were observed for the thyroid, while modest variations were observed for the heart and lungs. Only small variations were seen for the brainstem. The observed RBE variations within the patient population were similar for the organ mean dose-weighted RBE (${\overline{{\rm{RBE}}}}_{{\rm{d}}}$) and the effective near maximum dose RBE (RBE_D2%_). The two variable RBE models applied showed good agreement, with estimates of RBE consistently above 1.1. The results for the brainstem show that RBE variations among patients can be very small for a standardized treatment using the same beam configurations. In such cases applying a population based RBE value may be a step forward in treatment planning compared to using RBE of 1.1, as illustrated by the results in Fig. 4. These results also emphasize that the impact of inter-patient RBE variations must be evaluated in light of the physical organ doses, which in this study varied from mostly very low doses to the heart to high doses for the brainstem.

The observed inter-patient variations in RBE for the thyroid can be attributed to the spread in LET and dose between patients (Fig. 2d,h). The location of the thyroid distal to the spinal PTV can lead to significant doses deposited here, depending on the anatomy of the patient, i.e. the proximity of the thyroid to the target volumes. The margins applied to ensure dose coverage of the spinal PTV may also affect the dose and LET to the thyroid. The results presented in Fig. 4d, show that inter-patient variations in RBE does not necessarily lead to large differences between the RBE-weighted dose estimates based on the individual and population based RBE. In this particular case, the relatively low physical dose to the thyroid makes the RBE variations less critical.

The heart is also located distal to the PTV, but compared to the thyroid, the doses to the heart are low, and only a small fraction of the heart typically receives doses above 3–4 Gy(RBE). Therefore, the RBE_D2%_ parameter is of more relevance for the heart than the ${\overline{{\rm{RBE}}}}_{{\rm{d}}}$ parameter. Although some inter-patient variation in RBE_D2%_ was observed, the population median RBE_D2%_ values (Fig. 3b) give a reasonable representation of the RBE_D2%_ for each patient. The ${\overline{{\rm{RBE}}}}_{{\rm{d}}}$ and RBE_D2%_ parameters gave estimates of similar magnitude for the heart, due to the dose-weighting included in the ${\overline{{\rm{RBE}}}}_{{\rm{d}}}$ parameter. This demonstrates the robustness of this parameter compared to using the arithmetic mean of RBE values in all voxels. In principle, the OAR mean RBE could be calculated by simply averaging over RBE values on a voxel-by-voxel basis in the OAR of interest. However, for the purpose of evaluating RBE in a clinical setting, this approach could lead to artificially high RBE values in cases where the dose is inhomogeneous and low in parts of the organ. The low dose regions would then contribute to a significant increase in mean or median RBE as the RBE, for most models, is inversely proportional with dose^14^, and in addition, low dose regions often have high LET_d_ due to low energy scattered or secondary ions or neutrons. Considering the dose and LET_d_ distributions for the heart (Fig. 1) it is probable that the very high LET_d_ values observed in the LVH (Fig. 2e) originate from regions with negligible doses, mainly from secondary particles distal to the primary beam. Yet, LET_d_ values above 10 keV/µm were observed for the heart in regions receiving doses above the threshold of 0.5 Gy(RBE) illustrated in Fig. 1c. Overall, the ${\overline{{\rm{RBE}}}}_{{\rm{d}}}$ and RBE_D2%_ parameters gave similar values for all OARs. The slightly lower RBE_D2%_ observed for the heart and lungs could be due to lower LET in the high dose region (contributing more to RBE_D2%_ than ${\overline{{\rm{RBE}}}}_{{\rm{d}}}$), or due to the inverse dependence of RBE on dose, which in general gives a higher RBE in the low dose regions.

The inter-patient variations observed for the lungs were modest, resulting in only small deviations from the population median values (Fig. 3), and the agreement between RBE-weighted lung doses from individual and population RBE (Fig. 4c) was good for all patients. The dose deposition in the lungs is a result of lateral and distal margins as well as laterally scattered radiation and secondary particles, indicating the effect of anatomical differences. This leads to intermediate LET_d_ values and doses compared to the extremes seen for the heart (low dose, high LET_d_) and brainstem (high dose, low LET_d_).

The mean doses to the brainstem were similar for all patients (53–55 Gy(RBE_1.1_)), in combination with a relatively low LET_d_. These two factors contribute to low RBE values, and also relatively small variations in the brainstem RBE. However, contrary to the observations for the thyroid, the high doses deposited in the brainstem implies that even small changes in the RBE still could lead to non-negligible changes in RBE-weighted dose; the highest median RBE across the patient population (1.18 for both ${\overline{{\rm{RBE}}}}_{{\rm{d}}}$ and RBE_D2%_), resulted in an increase in estimated RBE-weighted mean dose of almost 4 Gy(RBE). The small degree of inter-patient RBE variations observed for the brainstem, in combination with consistent elevated RBE across the patient population, shows that the use of individual or population based RBE for the brainstem in CSI treatment planning may be advantageous compared to using the conventional RBE of 1.1. A recent review of brainstem injury from protons emphasizes the uncertainty in the proton RBE and the potential impact this may have on brainstem injury^24^. Brainstem injury incident rates are relatively low, and a clear connection between elevated RBE and brainstem injury has not yet been shown. However, across different institutions and treatment modalities (photons and protons) patients with brainstem necrosis received higher doses (median dose 54.7 Gy(RBE)) compared to those without brainstem injury (median dose 51.7 Gy(RBE)). With doses close to the clinical constraints reported by Haas-Kogan *et al*.^24^ (D_50%_ < 54 Gy(RBE), D_0.1cc_ < 58 Gy(RBE) and D_10%_ < 56 Gy(RBE)), the increase in RBE-weighted dose when moving to variable RBE (as seen in Table 1) illustrates that RBE effects could have a negative clinical impact for these patients.

The brainstem RBE values were similar to previously published results^15^; for 16 patients Giantsoudi et.al. found RBE values of 1.11–1.17 using the Frese model and 1.19–1.31 for the Carabe model. For the mean LET_d_ they found values in the range 2.5–3.9 keV/µm for the brainstem, which is somewhat higher compared to our study. It should, however, be noted that this study was performed using passively scattered proton therapy, which can result in different LET and RBE distributions compared to IMPT^22^. In addition, the brainstem LET_d_ can be dependent on other factors including field arrangements and target definitions^25^.

A previous study of two pediatric CSI patients (8 and 11 years) reports average RBE values (using MCN_(α/β)x 3Gy_) of 1.83 (heart), 1.58 (lungs) and 1.98 (thyroid)^16^. Using the same RBE model, our corresponding population median values for the heart (1.62) and lungs (1.27) were slightly lower. For the thyroid we observed a lower RBE of 1.44. Differences in RBE may be related to the obtained average (mean) dose of 1.5 Gy(RBE_1.1_) for the two patients compared to the median (mean) dose of 3.9 Gy(RBE_1.1_) across our patient population, again depending on patient anatomy, i.e. age/size of patient. Due to the small number of patients in the cited study, it is difficult to evaluate if the observed differences are due to inter-institution differences or inter-patient variations. The cited study also illustrated how different target definitions can affect the doses to OARs including the lungs, heart and thyroid. Traditionally, the whole vertebral column is included in the target volume for pediatric CSI patients to avoid the risk of spinal deformity related to an asymmetric dose across the vertebral body. This comes at the cost of increased doses to the OARs distal to the spinal target volume, and vertebral column sparing techniques are therefore an interesting option which potentially could lead to a clinical benefit for this patient group.

The tissue dependence of RBE for protons is included in most recent phenomenological RBE models. However, these parameters are associated with uncertainties both for the target regions as well as OARs. For the lungs, we adopted the (α/β)_x_ value of 4.0 Gy used by Öden *et al*.^20^ and based on the study by Bentzen *et al*.^26^. Radiation pneumonitis was the biological endpoint for these values, while e.g. late phase fibrosis (α/β)_x_ was suggested to be in the range of 2.0–3.0 Gy, also within the confidence intervals (CI) included in the present study. The brainstem (α/β)_x_ value is normally considered to be in the order of 2.0 Gy, although uncertainties are present also here^27^. For the heart and thyroid, a generic value for late effects of 3.0 Gy was applied^28^. Depending on the (α/β)_x_, the median ${\overline{{\rm{RBE}}}}_{{\rm{d}}}$ for the brainstem ranged from 1.15 to 1.22. Corresponding ranges for the heart and thyroid were 1.44–1.99 and 1.32–1.72. The uncertainties in the (α/β)_x_ values should therefore be carefully assessed both when evaluating the RBE values presented here and when estimating normal tissue complication probability for specific endpoints. The RBE changes monotonously with (α/β)_x_ for the ROR and MCN models^14^. The RBE for all (α/β)_x_ values within the confidence intervals will therefore fall within the shaded regions in Fig. 3. Thus, the RBE from other (α/β)_x_ values can be roughly assessed from the figures, and in principle, the variations in RBE with (α/β)_x_ could be determined analytically without new simulations.

While Wedenberg *et al*.^29^ showed that the (α/β)_x_ could be correlated to the RBE it is not clear if combining the α and β parameters into a single parameter is the best approach. Tissue parameters could also be handled separately, as in the proton RBE model by Jones *et al*.^30^. In radiosensitive tissue such as the brainstem and other parts of the central nervous tissue with low (α/β)_x_ the β parameter becomes increasingly important. RBE models differ significantly in how the RBE_min_ parameter (√β/β_*x*_) is implemented. While many models (including the ROR model) define the RBE_min_ as a constant of 1, there are RBE models with RBE_min_ both increasing^30,31^ and decreasing (including the MCN model) with LET. A recent study shows that the choice of dose range in the experimental *in vitro* data for the models may lead to different conclusions regarding the LET dependence of RBE_min_, and that data sets including low doses (below 1 Gy) indicate a slight increase in RBE_min_ with LET^32^. Overall, further investigations of the tissue dependency of the proton RBE are needed as the uncertainties in RBE associated with different tissues remains a significant challenge, complicating the introduction of variable RBE models in the clinical routine. Furthermore, when comparing different proton RBE models, derived from different experimental data, it is also clear that other uncertainties than the tissue dependence are present. Compared to other proton RBE models, the two models applied in this work showed intermediate RBE values^14^. One of the reasons for this may be that both the chosen models are based on large datasets from a number of different *in vitro* experiment, as opposed to many other proton RBE models.

Overall, this study demonstrates that for proton CSI treatments, OARs are subject to different degrees of inter-patient RBE variations. The observed RBE variations were reflected by the heterogeneity in dose and LET across the patients. Further RBE variations due to difference in radiosensitivity between patients (e.g. due to age) can, however, not be excluded. We also showed that the population based RBE values differ significantly from the clinically used value of 1.1. For OARs with small inter-patient variations in RBE, applying a population based RBE in treatment planning may therefore be a step forward compared to using an RBE of 1.1. Nevertheless, applying a constant RBE, although different from 1.1, has some of the same limitations as the current clinical practice as it ignores the variability in RBE within the organ. It should therefore be carefully evaluated if this approach gives disadvantages or advantages over other techniques accounting for a variable RBE such as LET-based optimization^33,34^. One limitation of purely LET-based optimization, compared to the population based approach presented here, is that it ignores the large variability in RBE with tissue type. Generally, OARs with large inter-patient RBE variations should be selected for patient-specific biological or RBE robustness analysis if the physical doses are close to known dose thresholds. Although proton CSI is a relatively standardized treatment with similar field and target configuration across different institutions, the variations in dose, LET and RBE across institutions could differ e.g. if alternative target definitions are used, and inter-institution differences should therefore be investigated further.

## Methods

IMPT plans for ten children (5–11 years) were generated using Eclipse (Varian Medical Systems, Palo Alto, CA, USA) applying RBE_1.1_ in the optimization. The planning target volume (PTV) included the cranio-spinal axis with vertebral bodies prescribed 23.4 Gy(RBE_1.1_) and a posterior fossa boost up to 54 Gy(RBE). Fraction doses were 1.8 Gy(RBE) for both the CSI and boost plans. Two posterior fields were applied to cover the spinal target volume while two lateral oblique opposing fields were applied the cranial target volume. Two additional lateral oblique opposing fields were applied to cover the boost volume. Optimization of the treatment plans was adapted from the standardized CSI treatment planning approach (including field angles and treatment planning margins) as described by Giebeler *et al*.^35^.

The plans were recalculated with the FLUKA Monte Carlo code^36,37^, obtaining the physical dose and LET_d_ on a voxel by voxel basis, as described in Fjæra *et al*.^25^. LET values were calculated based on primary and secondary protons. To estimate the RBE and RBE-weighted dose to the patients, two proton RBE-models were coupled to the Monte Carlo simulations. RBE was calculated based on the fraction doses. The models by McNamara *et al*. (MCN)^23^ and Rørvik *et al*. (ROR)^9^ include the LET, dose and (α/β)_x_ (fractionation sensitivity of photon treatments) as input parameters. The RBE was calculated using organ specific (α/β)_x_ values. For the heart, brainstem, lungs and thyroid, we applied (α/β)_x_ values of 3 (1.5–4.5) Gy^28^, 2.1 (1.1–3.2) Gy^38,39^, 4.0 (2.0–6.0)^20,26^ Gy and 3 (1.5–4.5) Gy^28^, respectively. Values in parenthesis are estimated 95% confidence intervals (CI) used to determine the impact of the tissue parameters and their uncertainty. The MCN model assume a linear relationship between LET and RBE and can therefore make use of the LET_d_ as input parameter while the ROR model is based on a non-linear LET-RBE relationship and requires the full LET spectrum to estimate the RBE.

Dose and LET_d_ volume histograms (DVH/LVH), RBE and mean and near maximum (D_2%_) values of RBE-weighted doses were estimated for important OARs: the heart, brainstem, lungs and thyroid. The D_2%_ parameter represent maximum dose received by 2% of the volume. From the RBE distribution and dose maps, the mean RBE for each organ was defined and calculated as the dose-weighted organ-mean RBE, ${\overline{{\rm{RBE}}}}_{{\rm{d}}}$:

1 $${\overline{{\rm{RBE}}}}_{d}=\frac{\frac{1}{N}{\sum }_{i=1}^{N}{{\rm{RBE}}}_{i}\times {D}_{phys,i}}{\frac{1}{N}{\sum }_{i=1}^{N}{D}_{phys,i}}=\frac{{\bar{D}}_{RBE}}{{\bar{D}}_{phys}},$$

where *N* is the total number of voxels in the organ and D_phys,i_ and RBE_i_ are the physical dose and RBE in voxel i. Equation (1) simplifies to the ratio of organ-mean RBE-weighted dose over organ-mean physical dose. This approach was chosen instead of calculating the arithmetic mean RBE on a voxel-by-voxel basis as the latter method will lead to less clinically relevant RBE values due to the high RBE in regions of negligible dose. In order to also evaluate the increase in D_2%_ from RBE effects a different approach must be used. In principle, the maximum RBE-weighted dose can be located in different voxels depending on whether a variable RBE model or RBE_1.1_ is used. In clinical evaluation of the treatment plans, the D_2%_ parameter is normally used without considering the location of the near maximum dose. In this study we therefore defined the effective maximum dose RBE (RBE_D2%_) as the ratio of D_2%_ for an RBE model and D_2%_ for the physical dose. There was no requirement of spatial correspondence between the two D_2%_ values as these where extracted from DVHs. RBE_D2%_ can then be calculated as:

2 $$RB{E}_{D2 \% }=\frac{{D}_{2 \% ,RBE}}{{D}_{2 \% ,phys}},$$

where D_2%,RBE_ is the near maximum RBE-weighted dose while D_2%phys_ is the near maximum physical dose. The RBE_D2%_ can then be used together with the physical dose to determine the increase in near maximum dose for a certain organ due to RBE effects.

From the ${\overline{{\rm{RBE}}}}_{{\rm{d}}}$ and RBE_D2%_ values, the population median RBE values were retrieved. In addition to the conventional RBE-weighted doses calculated with the patients individual RBE, the population median RBE values were applied to calculate RBE-weighted doses for all OARs and patients. To explore the usefulness of the population median RBE values, i.e. how well these values represent the RBE for individual patients, the population based RBE-weighted doses were compared to RBE-weighted doses calculated using the individual RBE values as well as doses calculated with RBE_1.1_. The population based RBE-weighted doses were calculated as:

3 $${\bar{D}}_{RBE}={\bar{D}}_{phys}\times {\overline{{\rm{RBE}}}}_{d,median},$$

where and ${\bar{D}}_{phys}$ is the mean dose for a specific OAR and patient, and ${\overline{{\rm{RBE}}}}_{d,median}$ is the median ${\overline{{\rm{RBE}}}}_{d}$ across the ten patients for this specific OAR.

## Supplementary information

Supplementary Information.
