# Supplementary material for: Inter-patient variations in relative biological effectiveness for cranio-spinal irradiation with protons
Source: Sci Rep. 2020 Apr 10;10:6212. doi: 10.1038/s41598-020-63164-8 (PMC7148381; doi:10.1038/s41598-020-63164-8)
Supplement: Supplementary file 1 — Supplementary Information. [file 41598_2020_63164_MOESM1_ESM.pdf]

Supplementary material

Inter-patient variations in relative biological effectiveness for cranio-spinal irradiation with protons

Kristian S. Ytre-Hauge[1*], Lars Fredrik Fjæra[1], Eivind Rørvik[2] , Tordis J. Dahle[1], Jon Espen Dale[3], Sara Pilskog[3] and Camilla H. Stokkevåg[3]

[1] Department of Physics and Technology, University of Bergen, Norway
[2] Department of Medical Physics, Oslo University Hospital, The Radium Hospital, Norway
[3] Department of Medical Physics and Oncology, Haukeland University Hospital, Norway

Figure S1 and Figure S2 compares doses from photon and proton treatment plans for the same patient as illustrated in Figure 1 in the main manuscript.

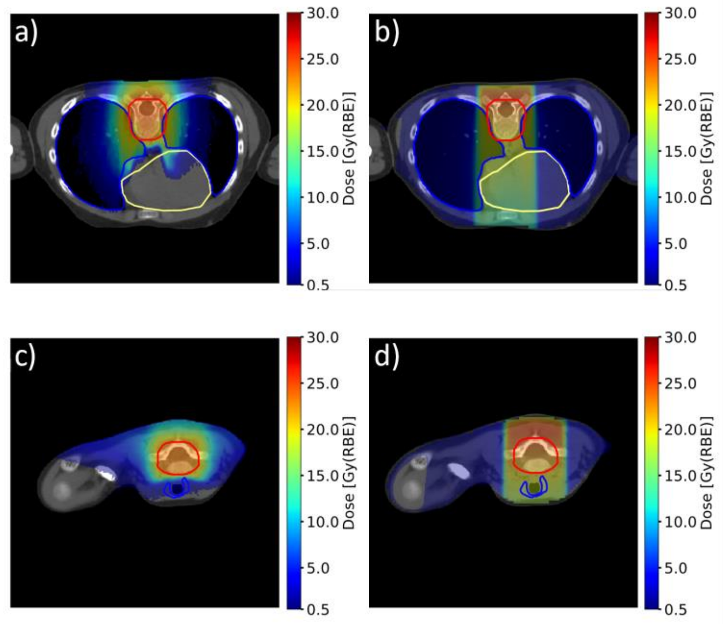

Figure S1: Dose distributions for intensity modulated proton therapy (IMPT) are shown in panel a) and c), while doses from photon treatment (3D-conformal radiotherapy) are shown in panel b) and d). 23.4 Gy(RBE) were prescribed to the target volumes. The IMPT plan was calculated using a constant RBE of 1.1. The spinal planning target volume, lungs, heart and thyroid are outlined in red, blue, yellow and blue, respectively.

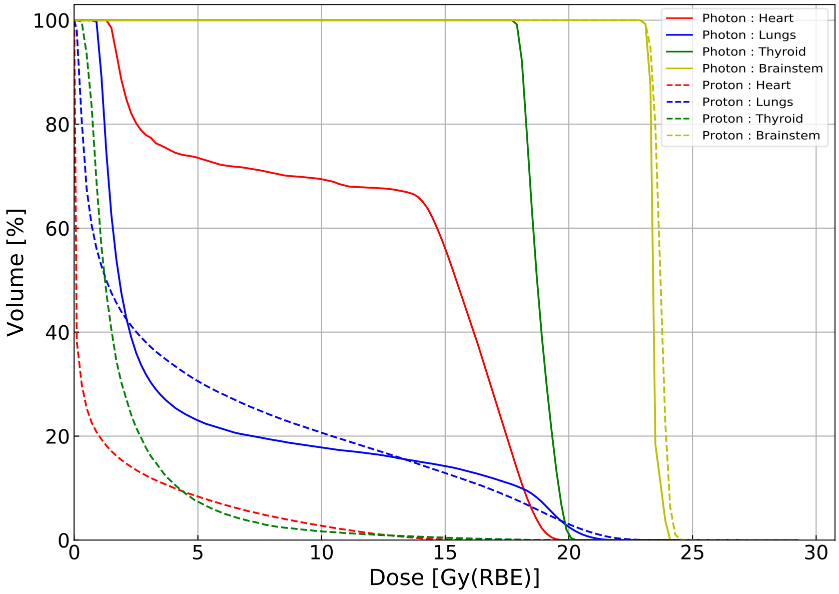

Figure S2: Dose volume histograms for the same patient as in Figure 1 and 1S are shown for the heart, lungs, thyroid and brainstem. Solid lines show the photon doses while dashed lines shows dose from protons calculated using a constant RBE of 1.1.
